# Knowledge, Beliefs and Preventive Practices Regarding Osteoporosis: A Cross-Sectional Study in Community Pharmacies in Tunis

**DOI:** 10.3390/nu17233759

**Published:** 2025-11-29

**Authors:** Cristina Merlan, Simona Buda, Alexandru Oancea, Narcisa Jianu, Teodor Nicolae Onea, Bianca Tot, Lucreția Udrescu, Vlad Groza, Mihai Udrescu, Adelina Lombrea, Denisa Maria Nițu, Alexandru Ciolofan, Farah Ben Jabeur, Cristina Adriana Dehelean, Valentina Oana Buda

**Affiliations:** 1 Faculty of Pharmacy, “Victor Babeş” University of Medicine and Pharmacy, 2 Eftimie Murgu Street, 300041 Timisoara, Romania; cristina.merlan@umft.ro (C.M.); bianca.tot@umft.ro (B.T.); udrescu.lucretia@umft.ro (L.U.); adelina.lombrea@umft.ro (A.L.); denisa.nitu@umft.ro (D.M.N.); farahbenjabeur53@gmail.com (F.B.J.); cadehelean@umft.ro (C.A.D.); buda.valentina@umft.ro (V.O.B.); 2 Doctoral School, “Victor Babeş” University of Medicine and Pharmacy, 300041 Timisoara, Romania; narcisa.dinu@umft.ro; 3 Research Center for Pharmaco-Toxicological Evaluation, “Victor Babeş” University of Medicine and Pharmacy, Eftimie Murgu Sq. No. 2, 300041 Timisoara, Romania; 4 Faculty of Medicine, “Vasile Goldiș” Western University of Arad, 81 Revolution Boulevard, 2900, 310025 Arad, Romania; 5 Politehnica University of Timişoara, Victoriei Square 2, 300006 Timisoara, Romania; teodornicolae.onea@gmail.com; 6 Center for Drug Data Analysis, Cheminformatics, and the Internet of Medical Things, “Victor Babeş” University of Medicine and Pharmacy, 300041 Timisoara, Romania; 7 Department of Computer and Information Technology, University Politehnica of Timişoara, 300223 Timisoara, Romania; vlad.groza@student.upt.ro (V.G.); mihai.udrescu@cs.upt.ro (M.U.); 8 Faculty of Medicine, “Victor Babeş” University of Medicine and Pharmacy, 2 Eftimie Murgu Street, 300041 Timisoara, Romania; ciolofan.alexandru@umft.ro; 9 Institute of Cardiovascular Diseases Timisoara, 13A Gheorghe Adam Street, 300310 Timisoara, Romania

**Keywords:** osteoporosis, public health, awareness, prevention, DXA, Tunisia, community survey

## Abstract

**Background/Objectives**: Osteoporosis represents a major yet underdiagnosed public health concern in developing countries, including Tunisia. Limited awareness, delayed diagnosis, and suboptimal adoption of preventive strategies contribute to increased risk of fragility fractures in aging populations. This study aimed to assess post-pandemic knowledge, attitudes, and preventive practices regarding osteoporosis in the Tunisian general population. **Methods**: A cross-sectional survey was conducted between February and April 2024 in six randomly selected community pharmacies in Tunis. Adults ≥ 40 years old completed a validated 31-item questionnaire assessing socio-demographic factors, osteoporosis knowledge, risk factors, screening practices, and preventive behaviors. A total of 160 fully completed questionnaires were analyzed using SPSS v22. **Results**: Only 60.6% of respondents reported awareness of osteoporosis. Knowledge levels were significantly higher in men (8.37 vs. 7.40; *p* = 0.043), urban residents (8.22 vs. 7.21; *p* = 0.036), participants with higher education (8.73 vs. 7.00; *p* < 0.001), those with a family history (8.58 vs. 7.49; *p* = 0.033), and individuals already diagnosed (9.19 vs. 7.63; *p* = 0.025). Screening rates were low: only 11.3% had ever undergone DXA testing, despite 18.8% reporting prior fractures. Preventive behaviors were inadequate: 80% did not supplement calcium, 88.1% did not use vitamin D, and 58.8% did not engage in <30 min of daily activity. Osteoporosis was reported by 13.1% of participants and 95.2% of diagnosed cases received treatment, predominantly bisphosphonates (75%). Comorbidities were significantly associated with osteoporosis (24.5% vs. 7.5%; *p* = 0.003). **Conclusions**: This study reveals suboptimal awareness, limited access to screening, and insufficient preventive behaviors regarding osteoporosis in Tunisia. Targeted educational initiatives, expansion of DXA availability, adoption of national osteoporosis guidelines, and multidisciplinary stakeholder involvement are critical to improve early detection, prevention, and management in the aging Tunisian population. Furthermore, promoting balanced nutrition that includes calcium- and vitamin D-rich foods, along with appropriate dietary supplementation when needed, is an essential preventive strategy to support optimal bone health and reduce osteoporosis risk in the general population.

## 1. Introduction

Osteoporosis is a systemic disease characterized by an imbalance between osteogenesis and bone resorption, which affects the microarchitecture of bone and increases the risk of fractures [1,2,3]. Depending on its etiology, osteoporosis can be divided into primary or secondary [4].

Primary osteoporosis usually has an involutional character as it occurs as a consequence of the ageing process; the most prevalent types of primary osteoporosis include postmenopausal (type I) and senile (type II) osteoporosis [4,5,6]. Type I osteoporosis is primarily caused by a sharp decline in estrogen and occurs in women after menopause, while type II osteoporosis affects both women and men after age 70 and it is characterized by reduced bone formation [6,7,8,9,10].

As life expectancy worldwide increases, the prevalence of debilitating diseases is expected to rise [1,11]. Osteoporosis, a global health concern, is one of the underdiagnosed and therefore undertreated debilitating diseases that often becomes obvious too late, when the first fragility fracture already occurred; consequently, its worldwide prevalence is still inaccurate today [11,12]. The best indicator for osteoporosis is the fracture rate in the older population [12]. The latest estimate of the prevalence of fragility fractures was published in 2000, reporting 56 million cases worldwide [11]. A systematic review reported an osteoporosis prevalence of 18.3% worldwide, with the highest prevalence, 39.5%, being recorded in Africa, although epidemiological studies in this continent are scarce [5]. On the national level, statistics are very concerning, as 16.2% of Tunisian women aged over 50 years old have a history of osteoporotic fracture, which suggests the lack of preventive measures and the delayed diagnosis and care of these patients [13]. The prevalence of osteoporosis is estimated to be even higher, at 23.4% of post-menopausal women [13,14,15].

Fragility fractures have a significant impact on the patient’s life, affecting their daily activities, leading to pain and deformities, thus reducing the quality of life and increasing mortality [6,16]. Among fragility fractures, hip fractures have the most serious consequences, with half of patients requiring permanent care after the first fracture [6,16,17,18]. Even though fragility fractures pose a significant public health issue, osteoporosis has not been a priority in Tunisia [14]. There is still a suboptimal level of diagnosis, prevention, and treatment, although screening of osteoporosis has been improved [19]. In Tunisia, similar to other Arab countries (i.e., Morocco, Algeria, Egypt), there is still a tremendous treatment gap between patients with a high risk of fractures and patients who receive adequate treatment for the prevention of osteoporotic fractures [14]. Multiple studies conducted in Arab countries revealed that over 90% of hip fracture patients were discharged without receiving any treatment for osteoporosis, and less than 5% of the patients were started on bisphosphonates [20].

Several guidelines currently provide updated recommendations on the prevention, diagnosis, evaluation, and treatment of osteoporosis, which can aid in clinical decision-making [14,17,18,21]. However, Tunisia lacks a national osteoporosis guideline. The Pan Arab Osteoporosis Society (PAOS) [22] and the African Society of Bone Health and Metabolic Diseases (ASBoM) provide recommendations for screening tools and treatment strategies that align with European guidelines. However, these recommendations are customized to address regional challenges, such as limited access to healthcare and modern diagnostic tools [14]. The most widely accepted test for diagnosing osteoporosis is the assessment of bone mineral density (BMD) using dual-energy X-ray absorptiometry (DXA) for the hip and lumbar spine [17,21,23]. DXA testing allows for the detection of decreased BMD, which is evaluated using the T-score [3]. In Tunisia, access to DXA testing for early diagnosis and treatment of osteoporosis is limited. This is partly due to the scarcity of DXA machines, which are mainly located in urban hospitals and private centers. Additionally, financial constraints are a significant factor, as patients must cover the costs themselves, and these expenses are not reimbursed [14,15].

Treatment is recommended for postmenopausal women and for men aged 50 years or more, and it should be chosen according to fracture risk [21,24]. The main drug classes used to treat osteoporosis are bisphosphonates, selective estrogen receptor modulators (SERMs), parathyroid hormone (PTH) analogs, RANK–ligand inhibitors, and sclerostin inhibitors [3,14,17]. Additionally, calcium and vitamin D supplementation at 1 g of calcium and 1000 IU of vitamin D is also advisable [11,18]. An online survey of Tunisian general practitioners and specialists performed in 2021 found that bisphosphonates are the preferred treatment for nearly all patients, with 99.8% of respondents selecting them. This preference can be partly attributed to socio-economic factors, as the national health insurance system reimburses this class of medication [25].

The world population is aging, and in Africa, the rise in life expectancy is estimated to be the most significant. The population aged 60 and above is expected to grow more than twice as fast in Africa as in Europe [14,26]. Given that the number of individuals aged 60 years and older will increase in the coming years and that osteoporosis prevalence is higher in older people, it is reasonable to expect an increase in the number of fragility fractures [1,11,27]. Elderly people with osteoporosis have an increased risk of falls, frailty, and complications due to comorbidities such as cardiovascular diseases (CVDs), obesity, and diabetes [28]. Knowing and addressing modifiable risk factors for osteoporosis enables people of all ages to improve their bone health from an early age. Also, identifying people at risk of developing osteoporosis is essential for early diagnosis and reducing fragility fractures and frailty [29,30,31].

Taking these facts into consideration, we intended to evaluate the level of knowledge among the Tunis population regarding osteoporosis and its risk factors. To the best of our knowledge, this survey represents the first study of its kind conducted in the last 5 years in this country. It serves as an exemplary model that elucidates deficiencies in osteoporosis awareness, thereby facilitating the identification of potential interventions to enhance prevention, diagnosis, and treatment.

## 2. Materials and Methods

### 2.1. Study Design

A cross-sectional, non-interventional study was conducted in six community pharmacies in Tunis over approximately 3 months, from 1 February to 30 April 2024. The objective was to assess the general population’s knowledge of osteoporosis in the Tunisian capital. A validated self-administered questionnaire was distributed to patients who visited the participating pharmacies during this time. A total of 160 fully completed questionnaires were included in the study.

The questionnaire consists of 31 items and was developed based on consultations with various European and Canadian sources in the scientific literature [32,33,34,35,36,37]. Further details about its structure and content can be found in our previously published paper [3]. In brief, the first section of the survey collected patients’ sociodemographic characteristics and their sources of information about osteoporosis. The following section aimed to evaluate the level of knowledge about osteoporosis among the Tunisian general population. This part included 13 dichotomous questions. Lastly, the questionnaire collected data on participants’ medical history, medication or dietary supplement use, dietary habits, and physical activity, along with a brief assessment of osteoporosis risk factors.

The questionnaire was formulated in both French and Arabic. Prior to distribution, it was pre-tested and validated by a commission of specialists using a three-stage method: pre-validation, constructive validation, and empirical validation. Firstly, a multidisciplinary team consisting of a clinical pharmacist, a general practitioner, a clinical pharmacy resident, and a pharmacy student composed the initial version of the questionnaire. Secondly, a team including a public health physician, an endocrinologist, and an orthopedist reviewed the questionnaire to evaluate its appropriateness from a clinical perspective. After the pre-validation phase, the questionnaire was administered to a small group of respondents (n = 8) to solicit feedback on its relevance, clarity, and degree of comprehension of the questions. Their recommendations were taken under advisement and were used to revise and update the questionnaire. Lastly, the revised questionnaire was distributed in 3 rural and three urban community pharmacies (n = 6 per pharmacy) to assess psychometric properties, namely test–retest reliability and internal consistency. A total of 44 respondents participated in the psychometric testing: 8 individuals in the preliminary pre-test and 36 respondents in the test–retest reliability assessment. Test–retest reliability was determined using the intraclass coefficient (ICC). We found an ICC value of 0.75 and an alpha coefficient of 0.7. These results indicate adequate internal consistency and homogeneity, thereby supporting the questionnaire’s validity.

### 2.2. Inclusion and Exclusion Criteria

All patients aged 40 years or older, able to read and write in French or Arabic, and willing to participate were included in the study. Patients under 40 years of age, those with cognitive disorders, those with language barriers, or those unwilling to participate were excluded from the study. Moreover, incompletely answered questionnaires were excluded from the analysis.

### 2.3. Pharmacy Selection Methodology and Sample Size Calculation

**Figure 1:**
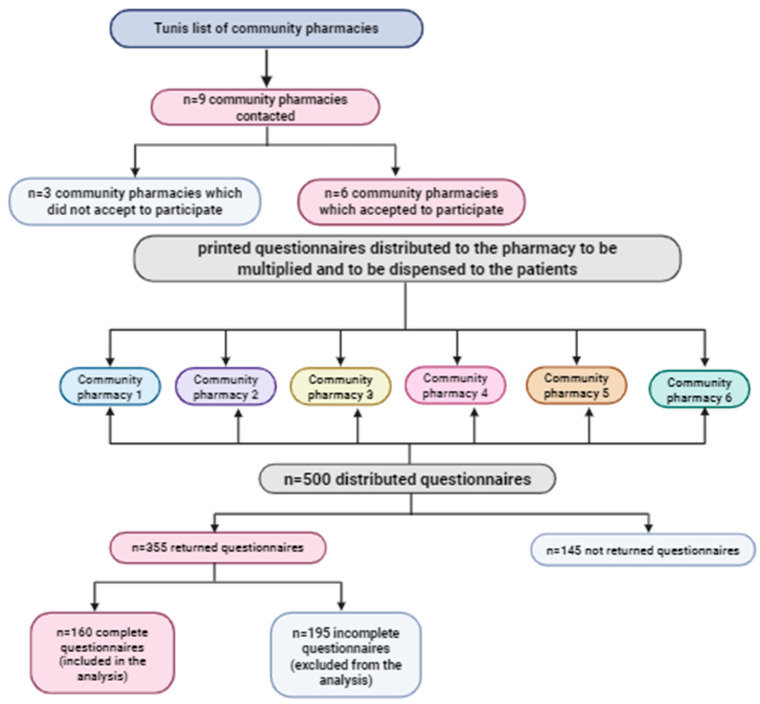
Flow diagram for study participants.

To eliminate bias, the process of pharmacy selection used a randomization algorithm applied to a complete list of community pharmacies in Tunis. Therefore, all pharmacies were randomly selected and included without regard to specific characteristics. Nine pharmacies were contacted; 6 accepted to participate and were included in the survey. Each participating pharmacy received printed questionnaires, which were then distributed for approximately 3 months. Of the 500 distributed questionnaires, 355 were returned by pharmacies, and only 160 were complete and therefore included in the study, as illustrated in Figure 1.

The sample size for this survey was determined using the standard formula for estimating a single proportion in a cross-sectional study. We assumed a conservative expected prevalence of 50% (maximum variability), a 95% confidence level (Z = 1.96), and an absolute precision (margin of error) of 8 percentage points. Under these assumptions, the minimum required sample size was approximately 150 participants. To allow for non-response and incomplete questionnaires, we distributed 500 paper forms across the six participating pharmacies. A total of 160 fully completed questionnaires were returned and included in the analysis, slightly exceeding the calculated target and corresponding to an effective margin of error of about 7–8% for key proportion estimates.

### 2.4. Data Collection

The pharmacies that agreed to participate in the survey designated two pharmacists to collect data. They informed the study participants about the study, offered the questionnaire, and responded to all participants’ questions. Moreover, they obtained written informed consent from all respondents and returned the completed questionnaires at the end of the established study period.

The obtained data were introduced into a Microsoft Excel document by two delegated persons. An identification number was assigned to each questionnaire to facilitate error checking. A third delegated person randomly reviewed and collected data to double-check its accuracy. Afterwards, body mass index (BMI) was calculated based on the anthropometric data obtained from the respondents: values < 18.5 kg/m^2^ correspond to underweight patients, values between 18.5–24.9 kg/m^2^ are considered normal, values between 25–29.9 kg/m^2^ correspond to overweight patients, and values > 30 kg/m^2^ are associated with obesity [36].

### 2.5. Data Analysis

For the statistical analysis of the collected data, the Statistical Package for the Social Sciences (SPSS) Version 22 (IBM, Armonk, NY, USA) was employed. A *p*-value lower than 0.05 was considered statistically significant. Data normality was evaluated using the Kolmogorov–Smirnov test. Categorical variables were all presented as numbers and percentages, while quantitative variables were presented as means and standard deviations. Comparisons between groups were performed using the Student *t*-test, ANOVA, and Chi-square test, as appropriate. Spearman’s correlation test was used to assess the relationship between the knowledge score and relevant variables. In addition to univariate comparisons, we performed a multiple linear regression analysis to explore independent correlations of osteoporosis knowledge. The total knowledge score (range 0–13 points) was used as a continuous dependent variable. Age, sex, level of education, place of residence (urban/rural), family history of osteoporosis and personal osteoporosis diagnosis were entered simultaneously as predictors. Model assumptions (linearity, homoscedasticity and normality of residuals) were checked and were considered acceptable. Results are presented as unstandardized regression coefficients (B) with 95% confidence intervals (CIs) and *p*-values.

### 2.6. Ethical Considerations

The study design complies with the Declaration of Helsinki and its amendments. Moreover, it received the approval of the Ethics Committee of the Victor Babes University of Medicine and Pharmacy (no. 47/2024). Written informed consent was attained from all study participants.

### 2.7. Analysis Using Complex Networks

In this study, we implement the methodology described in our previous article [3]. Specifically, we model our dataset as an undirected graph *G* = (*V*, *E*), where each node *v_i_* ∈ *V* symbolizes an individual participant, and each edge indicates a compatibility relationship between participants *i* and *j*. Using Mathematica 13.1’s energy-based layout, we cluster the network *G* to uncover communities of participants.

Within the anthropometric class, we discretize age into five 10-year intervals (40–49, 50–59, 60–69, 70–79, 80–89 years) and BMI into four categories (<18.5 kg/m^2^ underweight; 18.5–24.9 normal; 25.0–29.9 overweight; ≥30 obese); sex is binary (male/female). The demographic class includes discretized features: education—low/high—and environment—rural/urban. Lifestyle class consists of the following features: calcium supplementation, alcohol consumption, coffee intake, smoking status, and physical activity. The clinical class includes fracture history, comorbidities, osteoporosis diagnosis, DXA scan results, and osteoporosis treatment. All lifestyle and clinical features are binary (yes/no).

We set patient compatibility through a two-level procedure. The objective of edge construction was to encode overall phenotypic similarity rather than isolated feature matching. Therefore, we required concordance within each feature class and across multiple classes. In the first level, within each feature class, participants must match a minimum number of individual features (anthropometric ≥ 2/3, demographic 2/2, lifestyle ≥ 5/6, clinical ≥ 4/5). We selected these thresholds to reduce noise from individual items, avoid edges driven by a single dominant class, and preserve clinically meaningful resemblance. In the second level, globally, an edge is placed between two participants only if they satisfy at least three class-level criteria. The second-level criterion further limits spurious links by ensuring that connected participants are similar in several independent domains (body profile, socio-demographics, lifestyle behaviors, and clinical status). Together, these rules yielded well-connected graph enabling robust community detection.

**Figure 2:**
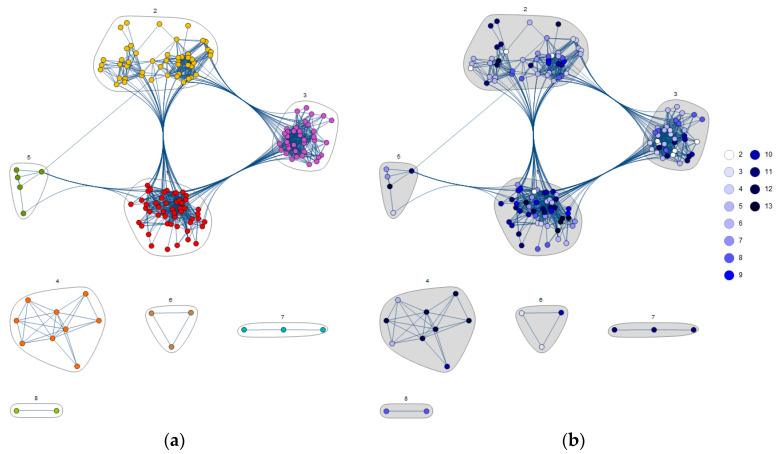
The undirected graph *G* of 157 participants (out of 160 total; three isolates have no edges), built based on pairwise compatibility and clustered using an energy-based layout. Clustering yields five communities within the principal connected component and three additional disconnected clusters (communities 4, 6, 7, and 8). (**a**) The color of the node indicates whether a participant belongs to a community. (**b**) The node colors encode each participant’s score in the questionnaire.

Our network analysis excludes the three node participants without edges; therefore, our investigation includes the 157 vertices/participants in communities 1–8 from Figure 2.

## 3. Results

The socio-demographic data of the Tunisian survey group are summarized in Table 1. Females accounted for 55.6% of the 160 respondents, while males accounted for 44.4% (n = 71). Most respondents were in the 50–59-year-old age group (30.0%), followed by those under 50 years (22.5%) and those aged 60–69 years (19.4%). A smaller proportion of participants were aged 70–79 years (15.0%), and 13.1% were aged 80 years or older. Regarding residency, the majority lived in urban areas (61.2%, n = 98), while 38.8% resided in rural areas. In terms of educational level, the largest proportion of participants had college-level education (54.1%), followed by primary school (26.8%) and high school (19.1%).

Table 2 presents the frequency of responses regarding knowledge and management of osteoporosis. A little over half of the respondents (60.6%) reported being knowledgeable about osteoporosis, while 39.4% reported not being aware of the condition. This shows that more than half of the surveyed population is aware of osteoporosis, although a significant portion remains unaware. Physicians were the primary source of information for 55.7% (n = 54) of the participants, emphasizing the important role of healthcare professionals in disseminating knowledge about osteoporosis. Other sources of information included family and friends (24.7%), social networks (14.4%), and media outlets like radio or television, which accounted for a small percentage (5.2%).

Regarding BMI, 46.9% of participants were classified as having normal weight, 31.9% as overweight, and 16.2% as obese. Only 5.0% of the respondents were underweight. This distribution suggests that nearly half of the population falls within a healthy weight range and only a small proportion is underweight, thus being predisposed to osteoporosis-related complications. However, other risk factors such as sedentarism and lack of prophylactic calcium and vitamin D supplementation contribute to a greater extent to osteoporosis prevalence.

Regarding falls, 81.2% of respondents reported no falls in the past year, while 18.8% did. Falls are an important risk factor for fractures, particularly in populations susceptible to osteoporosis. Additionally, 81.2% of the respondents reported no history of fractures, whereas 18.8% had previously experienced fractures, indicating a notable proportion of individuals at risk for osteoporosis-related fractures.

A large majority (80.0%, n = 128) of participants reported not taking calcium supplements, and 88.1% (n = 141) reported not taking vitamin D supplements, both of which are essential for bone health. Only 20.0% of the respondents reported taking calcium supplements, and 11.9% reported taking vitamin D supplements. These figures suggest a need for increased awareness and use of supplements to prevent osteoporosis.

Alcohol consumption was low among the respondents, with 93.7% reporting no alcohol use, 2.5% indicating occasional use, and 3.8% consuming alcohol regularly. Caffeine consumption was more common: 52.8% of respondents reported consuming caffeine, while 47.2% did not. Regarding smoking habits, 76.9% of the respondents were non-smokers, while 23.1% were active smokers (n = 37), indicating that smoking is still prevalent in a portion of the population, which could affect bone health.

Daily physical activity levels varied: 58.7% of participants reported less than 30 min of exercise per day, 30.0% reported 30–60 min, and 11.3% reported more than 60 min. Regular physical exercise is critical for maintaining bone health, and the data suggest that most respondents engage in lower levels of physical activity.

Family history of osteoporosis was reported by 31.3% of participants, indicating a genetic predisposition to the condition in nearly a third of the respondents. The majority (88.8%) had not undergone a DXA measurement of BMD, which is essential for diagnosing osteoporosis, while only 11.3% (n = 18) had taken the test. Furthermore, 86.9% of participants reported not having been diagnosed with osteoporosis, whereas 13.1% had been diagnosed (n = 21).

Among those diagnosed with osteoporosis, 95.2% reported receiving treatment, primarily with bisphosphonates (75.0%), followed by raloxifene (20.0%) and calcipotriol (5.0%). These treatment patterns reflect the standard therapeutic approaches to managing osteoporosis in the population.

We evaluated the impact of various socio-demographic and health-related factors on osteoporosis knowledge among Tunisian participants, our findings being presented in Table 3. Gender had a significant effect on knowledge levels, with males scoring higher than females (mean score 8.37 vs. 7.40, *p* = 0.043). This result contrasts with the expectation that females, being more at risk for osteoporosis, might have greater awareness.

In terms of age, the knowledge level did not differ significantly across age groups (*p* = 0.638), suggesting that age alone does not predict awareness of osteoporosis in this population. Participants aged 50–59 had the highest mean knowledge score (8.17), while those aged 70–79 had the lowest (7.12), but these differences were not statistically significant.

Residency was associated with osteoporosis knowledge, with urban residents demonstrating significantly higher levels than rural residents (mean score 8.22 vs. 7.21, *p* = 0.036). This suggests that individuals living in urban areas may have better access to information about osteoporosis, likely due to greater availability of healthcare services and greater media exposure.

Education level strongly influenced osteoporosis knowledge. Participants with college-level education scored significantly higher (mean score 8.73) than those with only primary school education (mean score 7.00) or high school education (mean score 6.60), with a *p*-value of 0.000, indicating that higher education is associated with greater awareness of osteoporosis.

Participants with a family history of osteoporosis also scored significantly higher in knowledge compared to those without a family history (mean score 8.58 vs. 7.49, *p* = 0.033), highlighting the impact of personal or familial health experiences on disease awareness.

A significant difference in knowledge was found between participants with and without an osteoporosis diagnosis (mean score 9.19 vs. 7.63, *p* = 0.025), suggesting that a personal diagnosis motivates individuals to seek more information about the disease.

Other factors, such as recent falls, BMI, and daily physical exercise, did not show a statistically significant effect on knowledge of osteoporosis (*p*-values > 0.05). This indicates that these variables do not strongly influence participants’ awareness or knowledge of the disease in this population. Additionally, no significant differences were found regarding the sources of information, although participants who received information from physicians had a higher mean knowledge score (9.17), compared to those who got information from family/friends (8.63) or social networks (7.79), though these differences were not statistically significant (*p* = 0.257).

In summary, gender, residency, education level, family history of osteoporosis, and personal diagnosis were significant predictors of osteoporosis knowledge, whereas age, BMI, recent falls, daily exercise, and source of information were not.

Table 4 presents a differential analysis of DXA test performance across different subject groups. In our study, the majority of participants (88.8%) reported that they had not undergone a DXA measurement of BMD, which is the primary diagnostic tool for osteoporosis. A total of 13 participants (8.1%) had undergone the test once, while 5 participants (3.1%) had taken it twice. Gender did not significantly affect DXA test uptake: 8.5% of males and 13.5% of females had undergone the test (*p* = 0.317). Additionally, there were no significant differences in DXA test uptake based on residency, education level, or recent falls. However, a significant relationship was found between having a history of fractures and DXA test uptake. A higher percentage (26.7%) of participants with a history of fractures had undergone the test, compared to only 7.7% of those without a history of fractures (*p* = 0.007). This indicates that a history of fractures is a key factor influencing whether individuals choose to undergo a DXA test.

Table 5 presents the differences in preventive practices and lifestyle behaviors between diagnosed and undiagnosed respondents. Among the total participants, 13.1% reported having an osteoporosis diagnosis, with the highest prevalence in the 70–79 age group (29.2%). Additionally, 16.7% of individuals aged 50–59 and 12.9% of those aged 60–69 were diagnosed with osteoporosis, while no cases were recorded in participants under the age of 50. Among those who engaged in less than 30 min of physical activity per day, 13.8% were diagnosed with osteoporosis. In contrast, none of the participants who exercised for more than 60 min daily had an osteoporosis diagnosis.

A significant association was found between taking calcium supplements and receiving an osteoporosis diagnosis (*p* = 0.000). Only 4.7% of participants without a diagnosis took calcium supplements, compared to 46.9% of those diagnosed with osteoporosis. Similarly, the use of vitamin D supplements was significantly higher among those with osteoporosis (73.7%) than in undiagnosed individuals (5.0%), with a *p*-value of 0.000. This indicates a strong correlation between osteoporosis diagnosis and the use of supplements aimed at supporting bone health.

We evaluated the impact of co-existing health conditions and the use of proton pump inhibitors (PPIs) on the prevalence of osteoporosis (results presented in Table 6). Participants with comorbidities, such as diabetes mellitus, thyroid disorders, kidney problems, or rheumatoid arthritis, exhibited a significantly higher prevalence of osteoporosis (24.5%) compared to those without these conditions (7.5%, *p* = 0.003). This indicates a strong association between comorbidities and the likelihood of developing osteoporosis. Additionally, respondents who had been on PPIs for more than three months showed a higher rate of osteoporosis diagnosis (20.0%) compared to those not on chronic PPI therapy (11.5%). However, this difference was not statistically significant (*p* = 0.234).

When examining falls, we found no significant difference in the incidence of recent falls between participants with and without comorbidities (*p* = 0.979). Nevertheless, PPI use for more than 3 months was associated with a higher prevalence of falls (30.0%) than in those not on long-term PPI therapy (16.2%), although this difference was not statistically significant (*p* = 0.080).

Regarding fracture history, participants with comorbidities had a higher, though not statistically significant, prevalence of fractures (24.5%) than those without comorbidities (15.9%; *p* = 0.188). The use of PPIs did not show a significant difference in fracture rates (*p* = 0.846). Overall, comorbidities emerged as a significant factor associated with osteoporosis prevalence, whereas PPI use showed trends that did not reach statistical significance.

To account for potential confounding between socio-demographic and clinical variables, we conducted a multiple linear regression with the osteoporosis knowledge score as the dependent variable. The overall model was statistically significant (F(6,150) = 4.31, *p* < 0.001), with an adjusted R^2^ of 0.113. In this multivariable analysis (Table 7), higher educational level and having a personal diagnosis of osteoporosis remained independently associated with higher knowledge scores (B = 0.89, 95% CI: 0.19–1.59, *p* = 0.013, and B = 1.60, 95% CI: 0.19–3.01, *p* = 0.027, respectively). Family history of osteoporosis showed only a borderline association (B = 0.91, *p* = 0.080). Age, sex and residency were no longer significant predictors after adjustment (all *p* > 0.05), indicating that the crude differences observed between men and women and between urban and rural residents are largely explained by differences in educational attainment and clinical exposure to osteoporosis.

As an exploratory complement to statistical analyses, we have conducted a complex network analysis to understand better the socio-demographic characteristics, clinical profiles, and behaviors of the communities comprising the general Tunis population. Figure 2 uses node coloring to indicate that each participant belongs to a community (panel a) and has an osteoporosis knowledge level score as assessed by our questionnaire (panel b).

Conventional tests (Table 3, Table 4, Table 5 and Table 6) identify associations between individual predictors and knowledge, DXA uptake, or diagnosis. The network analysis integrates these predictors simultaneously and partitions participants into multi-factor phenotypes. For example, Community 3 groups older, rural, overweight women with low supplementation and low physical activity, creating a profile that prioritizes prevention, whereas Community 4 identifies an urban, educated female subgroup with markedly higher fracture history and osteoporosis diagnosis, despite more frequent supplementation. These co-occurring patterns are not directly observable from univariate comparisons alone and provide a basis for tailoring public-health messages to distinct population types.

Communities 5–8 are small (n ≤ 5) and are reported for completeness. They suggest distinctive, hypothesis-generating phenotypes (e.g., underweight older women with diagnosis/treatment despite limited supplementation). Given their size, these communities should be interpreted cautiously and not as independent inferential findings.

Community 1, comprising 50 participants, is characterized by a markedly higher concentration of 40–49-year-old individuals (1.7 times the network average), a higher BMI (1.6), and a higher likelihood of holding a higher education degree (1.9) and residing in urban areas (1.6). Clinically, they exhibit a slightly elevated prevalence of comorbidities (1.2) but are marginally less likely to have been diagnosed with osteoporosis (1.1) or to be receiving osteoporosis treatment (1.1). Additionally, smoking is more common among these participants (1.4). No other features deviate meaningfully from the overall network percentage. We applied the same descriptive approach to each of the remaining communities.

Community 2 (57 participants) closely mirrors the overall network, with only a modest overrepresentation of 80–89-year-olds (1.6 times the average), those with lower education (1.3), and those without calcium supplementation (1.1). Clinically, they exhibit slightly higher proportions without an osteoporosis diagnosis (1.1) and osteoporosis treatment (1.1).

Community 3, comprising 37 participants, has a higher proportion of women (1.3 times the network average) and overrepresentation in the 70–79 (1.4) and 80–89 (1.8) age intervals. Members are overweight (1.5) and live in rural areas (2.6). In addition, members of this community do not take calcium supplements (1.2) and engage in low physical activity (1.1).

Community 4 (9 participants) is composed exclusively of women (1.8 times the network average) and has a strong representation of individuals in the 50–59 age range (2.8). Members exhibit a normal BMI (2.1), high educational attainment (1.9), and urban residency (1.3). Their lifestyle behavior includes calcium supplements (4.9), no smoking (1.3), and low physical activity (1.1). Clinically, this community shows elevated rates of fracture history (1.7), comorbidities (2.4), osteoporosis diagnosis (8.3), DXA scanning (2.9), and osteoporosis treatment (8.7).

Community 5, containing five male participants, is distinguished by participants primarily aged 60–69 (3.1 times the network average). These men are disproportionately underweight (8.9), reside in urban areas (1.6), and hold higher education degrees (1.9). They consume alcohol (7.9), smoke (4.4), and engage in over 60 min of daily physical activity (9.3).

Community 6 consists of three male participants and has a significant overrepresentation in the 70–79 age range (7.1 times the reference percentage). All members are of normal weight (2.1), take calcium supplements (4.9), and are non-smokers (1.3). Clinically, they have a history of fractures (1.7), a notable absence of comorbidities (1.5), osteoporosis diagnoses (8.3), DXA scans (5.8), and osteoporosis treatment (8.7).

Community 7, comprising three female participants, is notable for its high proportion of individuals aged 80–89 (5.0 times the network average). All members have normal BMI (1.4), live in rural areas (2.6), and display lower education (2.1). They commonly take calcium supplements (3.3) and exhibit comorbidities (3.1). Clinically, this community is diagnosed with osteoporosis (8.3), DXA-scanned (8.7), and receiving osteoporosis treatment (8.7).

Community 8, with two female participants, has characteristic features, including substantial overrepresentation in the 70–79-year-old age interval (7.1) and extreme underweight status (22.2). Neither member takes calcium supplements (1.3), and both lack comorbidities (1.5) or fracture history (1.7). Despite this, they are both diagnosed with osteoporosis (8.3), have undergone DXA scans (8.7), and are on osteoporosis treatment (8.7).

## 4. Discussion

Developing countries are characterized by higher osteoporosis rates, due to the lower socio-economic status of the general population, lower urbanization rates, impaired access to education, and to healthcare services [37]. Taking into account the phenomenon of population ageing, the osteoporosis rate is expected to grow at an accelerated rate, as in Tunisia, the number of elderly people is projected to double in the next 30 years, from 13.5% in 2020 to 25.4% in 2050, as reported by the United Nations Population Fund (UNFPA) [38]. Chronic non-communicable diseases are the main cause of disability in elderly people, of which musculoskeletal disorders are the most prevalent in women over 60 years old (21.4%) and the second most prevalent in men of the same age (18.1%) [38]. Of musculoskeletal disorders, osteoporosis has been directly linked to frailty: due to their common pathophysiological mechanisms, i.e., endocrine imbalance and chronic inflammation, the two conditions are interconnected [38]. Osteoporosis fractures contribute to frailty, chronic pain, loss of independence, and higher hospitalization and mortality rates, thus carrying a considerable burden to both the patient and the healthcare system [39]. Our study group aimed to evaluate the level of knowledge about osteoporosis and preventive strategies among the general population of Tunis to better understand the issue and contribute to the development of strategies to address it.

Regarding osteoporosis awareness, our findings are concerning: only 60.6% of respondents are aware of this condition. Interestingly, males had higher knowledge scores than women (mean score: 8.37 vs. 7.40, *p* = 0.043). These results contradict a previous study conducted by our group in Romania, which found that women scored higher than men [3]. This gender gap could be attributed in part to cultural differences between the two countries, because Tunisia is a Muslim state characterized by ideological conflicts between Western values (i.e., gender equality) and patriarchy [40]. However, previous studies did not find a positive correlation between religion and lower school education or between religion and negative health outcomes; on the contrary, Muslim religious practices have been associated with lower alcohol consumption, tobacco use, better mental health, and with preventive healthcare practices [41,42]. Unsurprisingly, respondents with a family history of osteoporosis or those diagnosed with osteoporosis showed better knowledge compared to those without a personal or family history of osteoporosis. These outcomes can be explained by the fact that the main sources of information for our study sample consist of physicians (55.7%), followed by family and friends (24.7%). Therefore, healthcare professionals are essential for disseminating scientifically accurate information about osteoporosis. Previous studies have shown that educational programs can have a significant impact on the implementation of prophylactic measures and on healthier habits among high-risk groups, underscoring the importance of educational awareness campaigns for shaping the health-related behavior of the population [43,44]. Moreover, we found that residency and educational level have a statistically significant influence on knowledge of osteoporosis in the general population. Our results are consistent with previous studies. Firstly, people living in urban areas have greater knowledge of osteoporosis than those in rural areas; these differences can be explained by easier access to information, greater availability of healthcare services, and exposure to social media [45,46]. Secondly, people with higher levels of education seem to be better informed about osteoporosis, probably due to greater access to information, educational programs, and preventive measures [37,47,48].

Another finding that should draw attention is that 9 out of 10 patients reported not having performed a DXA measurement of BMD. This is concerning because DXA is considered the standard in osteoporosis diagnosis, alongside clinical risk factors assessment, for all patients over 50 years old with osteoporosis risk factors or a history of falls [49,50]. As expected, we found a positive correlation between a history of fractures and DXA test uptake. The underuse of the diagnostic test can be justified by the insufficient availability of the devices in the region and by the high costs [50,51]. The unavailability of the DXA test should be addressed by healthcare policy makers, because this diagnostic device is essential for the early diagnosis and thus for increasing osteoporosis treatment rates [51]. Despite the impaired access to DXA testing, we found that 13.1% of respondents were diagnosed with osteoporosis, and 95.2% of them received treatment. Our findings are in accordance with other studies, which emphasize the undertreatment of this pathology, even in the presence of symptoms and fragility fractures [49]. Further studies are required in order to evaluate and address the osteoporosis treatment gap in the Tunisian population.

Comorbidities such as rheumatoid arthritis, metabolic diseases, and diabetes mellitus seem to increase the risk of developing osteoporosis. This can be explained by the fact that the treatment administered for these pathologies may decrease BMD, leading to osteopenia and osteoporosis, especially when administered chronically and in high dosages. Corticotherapy, frequently administered for rheumatic diseases or as a substitute treatment for adrenal insufficiency, has a negative effect on BMD, increasing the risk of osteopenia and osteoporosis [52,53]. Prescribing the lowest effective glucocorticoid dose could reduce the risk of secondary osteoporosis [52]. Cherif et al. have assessed the impact of the antidiabetic pharmacotherapy on BMD. This study included postmenopausal women treated for type 2 diabetes. Evaluation of BMD has shown that monotherapy with insulin increases the risk of fragility fractures, whereas metformin is associated with a safer profile [54]. Medication review services should be implemented to optimize pharmacotherapy and combat the overuse of osteoporosis-inducing medicines.

Our results highlight a lack of preventive behavior regarding osteoporosis. Calcium is considered an essential mineral for maintaining bone health and is frequently associated with vitamin D, which has a key role in calcium absorption and metabolism [55]. International guidelines recommend serum monitoring of vitamin D levels and, if necessary, supplementation with doses ranging from 10 μg (400 UI) to 50 μg (2000 UI), as nutrition and sunlight exposure alone are insufficient in the majority of patients and fail to maintain recommended serum vitamin D levels (30–50 nmol/liter) [14,56,57]. Additionally, ASBoM recommends, alongside vitamin D, calcium adjuvant therapy in doses of 1000 mg/day [14]. It is estimated that hypovitaminosis D (defined as serum vitamin D levels lower than 50 nmol/liter) has become a concerning public health issue due to its high prevalence in the Mediterranean region, Middle East and Africa, to its adverse events (i.e., decreased BMD, increased risk of fractures) and despite the sunny climate which should be favorable for the proper synthesis of vitamin D [55,58,59]. The countries most affected by vitamin D deficiency are predominantly Muslim nations, such as Algeria, Tunisia, and Morocco. Vitamin D deficiency rates in these countries are alarmingly high, with prevalence rates reaching 47.6% in Tunisia, 86% in Algeria, and 91% in Morocco. In contrast, European countries like Italy and Spain have lower osteoporosis rates, estimated at around 40% [55,56,57,58]. Several factors contribute to this phenomenon: (1) cultural and religious practices: many Muslim individuals wear more covering clothing, which limits sun exposure and reduces vitamin D synthesisꓼ (2) sunny climate: in very hot weather, people tend to avoid going outside, further decreasing sun exposureꓼ (3) sedentary lifestyle: there is a lack of outdoor exerciseꓼ (4) use of sunscreen formulation: the use of sun protection products that block ultraviolet B (UV-B) rays can hinder vitamin D synthesisꓼ (5) skin type: individuals with darker skin tones, which are richer in melanin, may require more sun exposure to produce adequate vitamin Dꓼ (6) inadequate diet: poor nutritional choices and insufficient supplement intake contribute to low vitamin D levelsꓼ (7) menopause timing: the onset of menopause tends to occur earlier in these populations, which can affect bone health [50,55,58,60]. In line with previous research, our findings indicate that approximately 80% of respondents do not take calcium and vitamin D supplements. Among those who do, a significant proportion are patients diagnosed with osteoporosis [47,61]. Consequently, we recommend calcium and vitamin D supplementation as supportive treatment, with less emphasis on their use as preventive measures. Moreover, the majority of participants reported insufficient physical activity: over half (approximately 58.8%) reported exercising for less than 30 min per day, while about one-third (around 30.0%) reported exercising 30 to 60 min per day. Sedentary behavior and lack of physical activity are associated with lower vitamin D levels, decreased bone mineralization, an increased risk of osteoporosis, and higher mortality rates [59,62,63].

The multivariable analysis provides additional insight into the determinants of osteoporosis knowledge in this cohort. After controlling for age, sex, residency and clinical factors, higher educational level and a personal diagnosis of osteoporosis emerged as the main independent correlates of better knowledge. Conversely, the apparent advantages observed in men and urban residents in the unadjusted analyses disappeared once education and diagnosis were taken into account. This pattern suggests that the gender- and residency-related differences in knowledge largely reflect underlying disparities in educational attainment and in prior contact with the healthcare system, rather than intrinsic effects of sex or place of residence. Nonetheless, the proportion of variance explained by the model was modest, indicating that unmeasured factors—such as health literacy, socioeconomic status or prior exposure to health campaigns—likely also play an important role.

In this study, complex network analysis was used as an exploratory complement to standard statistics. While inferential analysis quantify individual associations, complex network approach offers a robust framework for patient phenotyping by mapping multidimensional demographic, clinical, and lifestyle data into relational structures that highlight hidden, latent patterns and correlations [3]. This method reveals patient communities and identifies the most relevant phenotypic markers and actionable thresholds [64]. The largest communities aligned with the main statistical effects (education, residency, diagnosis), while smaller clusters are hypothesis-generating only. Moreover, complex network analysis can uncover community characteristics that conventional methods often overlook, thereby enabling more accurate stratification and targeted measures [65,66].

The eight communities reveal distinct participant profiles—ranging from younger, well-educated urban clusters with proactive calcium supplement use to elderly, rural groups with differing comorbidity burdens—underscoring the heterogeneity of osteoporosis risk factors and behaviors. These segmentation patterns suggest that targeted prevention and treatment strategies should focus on clinical, demographic, and lifestyle features that distinguish each community. However, due to their small sample sizes, the findings in communities 5–8 should be viewed as exploratory, yet they nonetheless underscore interesting, distinctive profiles (see Appendix A).

In summary, our findings highlight a significant knowledge gap among the general population of Tunis regarding osteoporosis. There is a pressing need for educational programs to improve understanding of the condition, implement more effective preventive strategies, and enhance access to healthcare services.

Integrated care based on the collaboration of primary care physicians, specialists, pharmacists, and other healthcare providers shows promising outcomes regarding the improvement of healthcare services [67]. Pharmacists and clinical pharmacists should closely collaborate with primary care physicians and specialists in order to develop educational programs for patients and high-risk groups to improve prevention strategies, diagnosis, and long-term management of osteoporosis [68,69].

Our findings should be interpreted in light of the current context in which we find ourselves. Coronavirus disease 2019 (COVID-19) has made its mark on healthcare, causing a delayed screening and treatment for acute and chronic diseases, including osteoporosis [70,71,72,73]. In recent years, many primary care facilities and clinics were temporarily closed or had disrupted programs, reducing the rate of DXA scans by more than 50%, thereby delaying diagnosis and treatment. Additionally, many patients did not have access to parenteral therapy, such as intravenous bisphosphonates. It has been reported in the literature that the administration of denosumab has decreased by about 18–49% after the onset of the COVID-19 pandemic [74]. 

To our knowledge, our work is the first attempt in the last 5 years to evaluate osteoporosis knowledge in the Tunis general population using a validated questionnaire, and it has the potential to serve as the cornerstone of broader, more extensive studies. The importance of our study lies in identifying knowledge gaps that can be effectively addressed through informative campaigns by authorities, in close cooperation with medical professionals. Clinical pharmacists should be deeply implicated in raising awareness about this pathology and its complications, which can increase the risk of frailty and decrease the quality of life of elderly patients. Moreover, our study offers a brief overview of the preventive measures and the treatment landscape in the Tunisian capital. Implementing a national osteoporosis guideline should be a priority for healthcare providers and policymakers to standardize the long-term management of this disease.

Nonetheless, our results should be interpreted with caution due to the study’s limitations. Firstly, the study design doesn’t allow for patient follow-up or for assessing the relationship between comprehension of osteoporosis and the evolution of the disease. Secondly, the sample size and the study period are relatively small. Although 500 questionnaires were distributed, only 160 fully completed forms were eligible for analysis. This completion rate reflects the practical challenges of administering a self-reported survey in busy community pharmacies. Moreover, low completion rate of the questionnaire could also be explained by the respondents’ hesitation to answer questions considered sensitive or personal and by the lack of incentives which could motivate the patients. While the final sample size remains close to the calculated minimum required for statistical validity, the limited response rate may affect the representativeness of the findings and restrict the extent to which results can be generalized to the broader adult population of Tunis. These factors should be considered when interpreting the conclusions. Thirdly, the sample bias: the recruitment of respondents was based on pharmacy visitors, which may exclude less mobile or rural populations and patients without regular access to pharmacies. Our findings are representative only of the study region and cannot be generalized to the entire country. Further studies are required to better understand osteoporosis as a public health issue in both rural and urban areas. It is worth mentioning that in our study the dietary behavior is inferred mainly from supplement use, without evaluation of dietary intake, food frequency, sunlight exposure or vitamin D status patterns. Lastly, the present study did not apply the Health Belief Model or Theory of Planned Behavior as its primary aim was to assess the osteoporosis-related level of knowledge by the means of a validated, non-theory-specific questionnaire. Designed as a cross-sectional study, our present work was not intended to predict behavioral outcomes or test theoretical constructs of health behavior. Furthermore, the selected instrument ensured content and construct validity for the current context without requiring linkage to a specific behavioral model. Future more extensive studies employing prospective, longitudinal or interventional designs will have to integrate also theoretical frameworks such as Health Belief Model or Theory of Planned Behavior to evaluate predictors of behavioral changes and to confirm potential causal pathways.

## 5. Conclusions

This cross-sectional survey highlights substantial gaps in osteoporosis awareness, prevention, and screening among the general population of Tunis. Although approximately 61% of respondents reported familiarity with osteoporosis, overall knowledge levels remained insufficient, particularly among individuals with lower educational attainment and those residing in rural areas. Access to diagnostic services was markedly limited, with only ~11% of participants having undergone DXA scanning, underscoring both structural and financial barriers to early detection.

Preventive practices were notably suboptimal; the majority of respondents did not engage in adequate physical activity nor use calcium or vitamin D supplements, and prevention behaviours were predominantly adopted only after diagnosis rather than proactively. Despite low diagnosis rates (~13%), treatment adherence among diagnosed patients was high, indicating that once osteoporosis is identified, therapeutic management is generally appropriate. Comorbidities including kidney problems, thyroid disorders, diabetes mellitus and rheumatoid arthritis were significantly associated with osteoporosis, emphasizing the need for targeted screening strategies in high-risk groups.

Overall, these findings underscore an urgent need for structured national strategies to improve public and professional awareness, expand access to diagnostic resources, and promote evidence-based preventive behaviours. Implementing national osteoporosis guidelines, fostering multidisciplinary collaboration among healthcare providers, and launching public health campaigns may help reduce the burden of osteoporosis, prevent fragility fractures, and improve long-term outcomes in the aging Tunisian population.

In addition, strengthening public education on balanced nutrition—with emphasis on regular consumption of calcium- and vitamin D-rich foods, and supplementation when dietary intake is insufficient—should represent a core component of national osteoporosis prevention strategies, supporting bone health and reducing long-term fracture burden.

## Figures and Tables

**Table 1:** The absolute (count) and relative (%) frequency of socio-demographic characteristics.

Variable	Group	Count	%
Gender	Male	71	44.4%
	Female	89	55.6%
Age range	<50	36	22.5%
	50–59	48	30.0%
	60–69	31	19.4%
	70–79	24	15.0%
	>80	21	13.1%
Residency	Rural	62	38.8%
	Urban	98	61.2%
Level of education	Primary school	42	26.8%
	High-school	30	19.1%
	College	85	54.1%

**Table 2:** The absolute (count) and relative (%) frequency of responses corresponding to osteoporosis signs, symptoms, risk factors, family history, prevention, diagnosis, management, and treatment.

Variable	Group	Count	%
Knowledge about osteoporosis	No	63	39.4%
	Yes	97	60.6%
Source of information	Physician	54	55.7%
	Family/friends	24	24.7%
	Social networks	14	14.4%
	Others (radio/TV)	5	5.2%
BMI	Underweight (<18.5 kg/m^2^)	8	5.0%
	Normal weight (18.5–25 kg/m^2^)	75	46.9%
	Overweight (25–30 kg/m^2^)	51	31.9%
	Obesity (>30 kg/m^2^)	26	16.2%
Recent falls (in the last year)	No	130	81.2%
	Yes	30	18.8%
History of fractures	No	130	81.2%
	Yes	30	18.8%
Taking calcium supplements	No	128	80.0%
	Yes	32	20.0%
Taking vitamin D supplements	No	141	88.1%
	Yes	19	11.9%
Alcohol consumption	No	150	93.7%
	Occasionally	4	2.5%
	Yes	6	3.8%
Caffeine consumption	No	75	47.2%
	Yes	84	52.8%
Active smoker	No	123	76.9%
	Yes	37	23.1%
Daily physical exercises	<30 min/day	94	58.7%
	30–60 min/day	48	30.0%
	>60 min/day	18	11.3%
Family history of osteoporosis	No	110	68.7%
	Yes	50	31.3%
DXA measurement of BMD	No	142	88.7%
	Yes	18	11.3%
Osteoporosis diagnosis	No	139	86.9%
	Yes	21	13.1%
Osteoporosis treatment	No	1	4.8%
	Yes	20	95.2%
Type of treatment	Bisphosphonates	15	75.0%
	Raloxifene	4	20.0%
	Calcipotriol	1	5.0%

**Table 3:** Differential analysis of osteoporosis knowledge level scores in groups of subjects.

Variable	Group	Count	Mean of Knowledge Level	Std. Deviation of Knowledge Level	*p*-Value
Gender	Male	71	8.37	2.75	0.043 *
	Female	89	7.40	3.13	
Age range	<50	36	7.86	3.05	0.638
	50–59	48	8.17	3.17	
	60–69	31	8.10	2.88	
	70–79	24	7.12	3.19	
	>80	21	7.43	2.46	
Residency	Rural	62	7.21	3.21	0.036 *
	Urban	98	8.22	2.79	
Level of education	Primary school	42	7.00	2.96	0.000 *
	High-school	30	6.60	2.70	
	College	85	8.73	2.89	
Recent falls (in the last year)	No	130	7.77	2.91	0.587
	Yes	30	8.10	3.38	
Family history of osteoporosis	No	110	7.49	3.03	0.033 *
	Yes	50	8.58	2.81	
Osteoporosis diagnosis	No	139	7.63	2.88	0.025 *
	Yes	21	9.19	3.46	
BMI	Underweight (<18.5 kg/m^2^)	8	7.63	2.62	0.972
	Normal weight (18.5–25 kg/m^2^)	75	7.92	2.97	
	Overweight (25–30 kg/m^2^)	51	7.84	3.37	
	Obesity (>30 kg/m^2^)	26	7.62	2.50	
Daily physical exercises	<30 min/day	94	7.86	3.01	0.712
	30–60 min/day	48	7.60	3.09	
	>60 min/day	18	8.28	2.74	
Source of information	Physician	54	9.17	2.93	0.257
	Family/friends	24	8.63	2.45	
	Social networks	14	7.79	3.64	
	Others (radio/TV)	5	7.20	2.39	

Legend: *—statistical significance.

**Table 4:** Differential analysis of DXA test performance in groups of subjects.

Variable	Group	DXA Test = No		DXA Test = Yes		*p*-Value
		Count	%	Count	%	
Gender	Male	65	91.5%	6	8.5%	0.317
	Female	77	86.5%	12	13.5%	
Residency	Rural	57	91.9%	5	8.1%	0.310
	Urban	85	86.7%	13	13.3%	
Level of education	Primary school	37	88.1%	5	11.9%	-
	High-school	27	90.0%	3	10.0%	
	College	75	88.2%	10	11.8%	
Recent falls (in the last year)	No	117	90.0%	13	10.0%	0.336
	Yes	25	83.3%	5	16.7%	
History of fractures	No	120	92.3%	10	7.7%	0.007 *
	Yes	22	73.3%	8	26.7%	

Legend: *—statistical significance.

**Table 5:** Differences in preventive practices and lifestyle behaviors between diagnosed and undiagnosed respondents.

Variable	Group	Osteoporosis Diagnosis = No		Osteoporosis Diagnosis = Yes		*p*-Value
		Count	%	Count	%	
Age range	<50	36	100.0%	0	0.0%	-
	50–59	40	83.3%	8	16.7%	
	60–69	27	87.1%	4	12.9%	
	70–79	17	70.8%	7	29.2%	
	>80	19	90.5%	2	9.5%	
Daily physical exercises	<30 min/day	81	86.2%	13	13.8%	-
	30–60 min/day	40	83.3%	8	16.7%	
	>60 min/day	18	100.0%	0	0.0%	
Taking calcium supplements	No	122	95.3%	6	4.7%	0.000 *
	Yes	17	53.1%	15	46.9%	
Taking vitamin D supplements	No	134	95.0%	7	5.0%	0.000 *
	Yes	5	26.3%	14	73.7%	

Legend: *—statistical significance.

**Table 6:** The effect of associated pathologies and the use of proton pump inhibitors on osteoporosis diagnosis and risk factors.

Variable	Group	Response = No		Response = Yes		*p*-Value
		Count	%	Count	%	
**Osteoporosis diagnosis**						
Comorbidities	No	99	92.5%	8	7.5%	0.003 *
	Yes	40	75.5%	13	24.5%	
PPI administration (>3 months)	No	115	88.5%	15	11.5%	0.234
	Yes	24	80.0%	6	20.0%	
**Recent falls**						
Comorbidities	No	87	81.3%	20	18.7%	0.979
	Yes	43	81.1%	10	18.9%	
PPI administration (>3 months)	No	109	83.8%	21	16.2%	0.080
	Yes	21	70.0%	9	30.0%	
**History of fractures**						
Comorbidities	No	90	84.1%	17	15.9%	0.188
	Yes	40	75.5%	13	24.5%	
PPI administration (>3 months)	No	106	81.5%	24	18.5%	0.846
	Yes	24	80.0%	6	20.0%	

Legend: PPI—proton pump inhibitors, *—statistical significance.

**Table 7:** Multiple linear regression analysis of factors associated with osteoporosis knowledge score.

Variable	Coefficient	Std. Error	t-Statistic	*p*-Value
(Constant)	5.551	1.930	2.876	0.005 *
Age	−0.001	0.021	−0.047	0.962
Gender	−0.583	0.524	−1.113	0.267
Level of education	0.890	0.353	2.523	0.013 *
Residency	0.275	0.507	0.542	0.589
Family history of osteoporosis	0.909	0.516	1.762	0.080
Osteoporosis diagnosis	1.597	0.714	2.235	0.027 *
Model summary: R^2^ = 0.147; adjusted R^2^ = 0.113; F-statistic = 4.309, *p* < 0.001 *				

Legend: *—statistical significance.

## Data Availability

The original contributions presented in this study are included in the article and in the Supplementary Materials. Further inquiries can be directed to the corresponding authors.

## Supplementary Materials

The following supporting information can be downloaded at: https://www.mdpi.com/article/10.3390/nu17233759/s1, Questionnaire Q1: Osteoporosis Questionnaire Table.

## Abbreviations

The following abbreviations are used in this manuscript:

ASBoM	African Society of Bone Health and Metabolic Diseases
BMD	Bone mineral density
BMI	Body mass index
COVID-19	Coronavirus disease 2019
CVD	Cardiovascular disease
DXA	Dual-energy X-ray absorptiometry
ICC	Intraclass coefficient
PAOS	Pan Arab Osteoporosis Society
PPI	Proton pump inhibitor
PTH	Parathyroid hormone
SARS-CoV-2	Severe acute respiratory syndrome coronavirus 2
SERM	Selective estrogen receptor modulator
SGLT-2	Sodium-glucose cotransporter-2
SSRI	Selective serotonin-reuptake inhibitor
UNFPA	United Nations Population Fund
UV-B	Ultraviolet B

## Appendix A

**Table A1:** The patient features and their distribution across the eight communities: the first two columns list each feature’s name, classification level, and the total number of participants exhibiting that feature. Subsequent columns report, for each community, the count and the relative proportion of members possessing that feature.

Feature	Feature’s Interval/Level (*n*, Number of Participants)	Community 1 *(n* = 50)	Community 2 (*n* = 48)	Community 3 (*n* = 37)	Community 4 (*n* = 9)	Community 5 (*n* = 5)	Community 6 (*n* = 3)	Community 7 (*n* = 3)	Community 8 (*n* = 2)
Age (years)	40–49 (35)	19 (1.7)	7 (0.7)	0 (0)	0 (0)	0 (0)	0 (0)	0 (0)	0 (0)
	50–59 (49)	18 (1.2)	17 (1.1)	4 (0.3)	8 (2.8)	2 (1.3)	0 (0)	0 (0)	0 (0)
	60–69 (30)	9 (0.9)	8 (0.9)	8 (1.1)	1 (0.6)	3 (3.1)	0 (0)	1 (1.7)	0 (0)
	70–79 (22)	4 (0.6)	6 (0.9)	7 (1.4)	0 (0)	0 (0)	3 (7.1)	0 (0)	0 (0)
	80–89 (21)	0 (0)	10 (1.6)	9 (1.8)	0 (0)	0 (0)	0 (0)	2 (5)	0 (0)
Sex	Males (69)	27 (1.2)	25 (1.2)	9 (0.6)	0 (0)	5 (2.3)	3 (2.3)	0 (0)	0 (0)
	Females (88)	23 (0.8)	23 (0.9)	28 (1.3)	9 (1.8)	0 (0)	0 (0)	3 (1.8)	2 (1.8)
BMI (kg/m^2^)	≤18.49 (7)	0 (0)	3 (1.4)	0 (0)	0 (0)	2 (8.9)	0 (0)	0 (0)	2 (22.2)
	18.5–24.99 (76)	19 (0.8)	28 (1.2)	13 (0.7)	9 (2.1)	2 (0.8)	3 (2.1)	2 (1.4)	0 (0)
	25–29.99 (50)	25 (1.6)	5 (0.3)	18 (1.5)	0 (0)	1 (0.6)	0 (0)	1 (1.0)	0 (0)
	≥30 (24)	6 (0.8)	12 (1.6)	6 (1.1)	0 (0)	0 (0)	0 (0)	0 (0)	0 (0)
Education level	Low (74)	0 (0)	29 (1.3)	37 (2.1)	0 (0)	0 (0)	3 (2.1)	3 (2.1)	3 (2.1)
	High (83)	50 (1.9)	19 (0.7)	0 (0)	9 (1.9)	5 (1.9)	0 (0)	0 (0)	0 (0)
Living environment	Urban (96)	50 (1.6)	29 (1.0)	0 (0)	7 (1.3)	5 (1.6)	3 (1.6)	0 (0)	2 (1.6)
	Rural (61)	0 (0)	19 (1.0)	37 (2.6)	2 (0.6)	0 (0)	0 (0)	3 (2.6)	0 (0)
Calcium supplements	Yes (32)	9 (0.9)	6 (0.6)	3 (0.4)	9 (4.9)	0 (0)	3 (4.9)	2 (3.3)	0 (0)
	No (125)	41 (1.0)	42 (1.1)	34 (1.2)	0 (0)	5 (1.3)	0 (0)	1 (0.4)	2 (1.3)
Alcohol consumption	Yes (6)	3 (1.6)	0 (0)	0 (0)	0 (0)	3 (7.9)	0 (0)	0 (0)	0 (0)
	No (151)	47 (1.0)	48 (1.0)	9 (1.0)	2 (0.4)	3 (1.0)	3 (1.0)	3 (1.0)	2 (1.0)
Smoking	Yes (36)	16 (1.4)	11 (1.0)	4 (0.5)	0 (0)	5 (4.4)	0 (0)	0 (0)	0 (0)
	No (121)	34 (0.9)	37 (1.0)	33 (1.2)	9 (1.3)	0 (0)	3 (1.3)	3 (1.3)	2 (1.3)
Daily physical activity	≤60 min (140)	42 (0.9)	44 (1.0)	37 (1.1)	9 (1.1)	0 (0)	3 (1.1)	3 (1.1)	2 (1.1)
	>60 min (17)	8 (1.5)	4 (0.8)	0 (0)	0 (0)	5 (9.3)	0 (0)	0 (0)	0 (0)
Fracture history	Yes (62)	16 (0.8)	18 (0.9)	16 (1.1)	6 (1.7)	3 (1.5)	2 (1.7)	1 (0.8)	0 (0)
	No (95)	34 (1.1)	30 (1.0)	21 (0.9)	3 (0.6)	2 (0.7)	1 (0.6)	2 (1.1)	2 (1.7)
Comorbidities	Yes (51)	10 (0.6)	17 (1.1)	13 (1.1)	7 (2.4)	1 (0.6)	0 (0)	3 (3.1)	0 (0)
	No (106)	40 (1.2)	31 (1.0)	24 (1.0)	2 (0.3)	4 (1.2)	3 (1.5)	0 (0)	2 (1.5)
Diagnosis of osteoporosis	Yes (19)	0 (0)	1 (0.2)	1 (0.2)	9 (8.3)	0 (0)	3 (8.3)	3 (8.3)	2 (8.3)
	No (138)	50 (1.1)	47 (1.1)	36 (1.1)	0 (0)	5 (1.1)	0 (0)	0 (0)	0 (0)
DXA	Yes (18)	7 (1.2)	1 (0.2)	2 (0.5)	3 (2.9)	0 (0)	2 (5.8)	3 (8.7)	0 (0)
	No (139)	43 (1.0)	47 (1.1)	35 (1.1)	6 (0.8)	5 (1.1)	1 (0.4)	0 (0)	2 (1.1)
Osteoporosis treatment	Yes (18)	0 (0)	1 (0.2)	0 (0)	9 (8.7)	0 (0)	3 (8.7)	3 (8.7)	2(8.7)
	No (139)	50 (1.1)	47 (1.1)	37 (1.1)	0 (0)	5 (1.1)	0 (0)	0 (0)	0 (0)
