# Supplementary material for: Knowledge, Beliefs and Preventive Practices Regarding Osteoporosis: A Cross-Sectional Study in Community Pharmacies in Tunis
Source: Nutrients. 2025 Nov 29;17(23):3759. doi: 10.3390/nu17233759 (PMC12694441; doi:10.3390/nu17233759)
Supplement: Supplementary file 1 [file nutrients-17-03759-s001.zip › nutrients-3995923-supplementary.pdf]

OSTEOPOROSIS QUESTIONNAIRE

Dear patient,

Thank you for your agreement to complete this questionnaire! (Duration: maximum 5 minutes)

This questionnaire is intended for people over the age of 40 and will be used to conduct study on the population's knowledge and awareness of osteoporosis and its risk factors.

Your participation is **voluntary**, and the data and **information** provided will be strictly **confidential**.

1. Age: .... years old **2. Sex:** □ Woman □ Man **3. Height:** .... cm. **4. Weight:** .... kg.

5. Last level of studies completed:

□ Elementary school □ Highschool □ University □ Other studies: ....

6. You live in: □ rural area □ urban area

7. Your general practitioner lives in: □ rural area □ urban area

8. Do you know any information about osteoporosis? □ Yes □ No

9. From where/from whom? (What sources of information did you use?)

□ Friends/family □ Doctor (□ general practitioner/□ specialist) □ Social networks (e.g., Facebook)

□ Other sources (e.g., radio, TV)

10. Select "Yes" or "No" to indicate your knowledge of the following osteoporosis risk factors:

a) A family history of osteoporosis strongly predisposes a person to this disease □ Yes □ No

b) Osteoporosis is more common in women than in men □ Yes □ No

c) There is a small loss of bone mass in the first 10 years after the onset of menopause □ Yes □ No

d) Osteoporosis increases the risk of bone fractures □ Yes □ No

e) Osteoporosis causes symptoms (e.g., pain) before possible fractures occur □ Yes □ No

f) When it comes to risk of fractures, a fall is as important as having low bone density □ Yes □ No

g) Starting at age 50, most women can expect at least one fracture in the next few years □ Yes □ No

h) Smoking can contribute to osteoporosis □ Yes □ No

i) High salt intake is a risk factor for osteoporosis □ Yes □ No

j) Any type of physical activity can help reduce the risk of developing osteoporosis □ Yes □ No

k) An adequate intake of calcium can be obtained by drinking 2 glasses of milk per day □ Yes □ No

l) Moderate alcohol consumption has negative effects on the onset of osteoporosis □ Yes □ No

m) There are currently effective treatments for osteoporosis □ Yes □ No

11. Depending on your gender, please answer the following questions:

For women:	For men:
a. At what age did you have your first period?<br>.......... years old<br>b. Are you still having menstrual periods?<br>□ Yes □ No<br>c. At what age did you last have your period?<br>.......... years old<br>e. Have you ever had irregular periods?<br>□ Yes □ No<br>f. Do you have any information regarding hormone replacement therapy during menopause?<br>□ Yes □ No<br>If yes, from whom?	a. Do you have low testosterone levels?<br>□ Yes □ No<br>b. Do you have low libido?<br>□ Yes □ No<br>c. Do you have any prostate problems?<br>□ Yes □ No<br>c1. If yes, for how long?<br>.......... years<br>c2. If yes, are you taking any treatment?<br>□ Yes □ No<br>If yes, who recommended it to you? ....

12. Do you have any of the following problems?

□ Balance problems □ Dizziness □ Vision problems □ Not the case

13. Have you experienced any recent falls (in the last year)? □ Yes No

If yes, the consequences were: □ minor □ major (e.g., fractures)

14. Have you ever had bone fractures in the past? □ Yes □ No

If yes, what kind of fractures? □ Hip □ Pelvis □ Wrist □ Shoulder □ Spine

□ Other kind of fractures: ....

If yes, how many times? ....

15. The fracture occurred as a result of:

□ falls from leg height (e.g., tripping, slipping, falling out of bed)

□ hard falls (e.g., from a ladder, from a bicycle, or by rolling down stairs)

□ car accidents or other major traumas

□ Not the case

16. Do you have a family history of fractures? □ Yes □ No

If yes, who suffered the fracture? ....

17. How much calcium do you get from your diet each day? (for instance, a glass of milk: 300 mg of Calcium, an orange: 43 mg, a cup of plums: 75 mg, 180 g beans: 130 mg, a can of 85 g salmon: 210 mg)

□ <500 mg □ >500 mg □ I'm not sure □ Not applicable (e.g., lactose or citrus intolerance)

18. Do you take any calcium supplements? □ Yes □ No

If yes, how much? ....

If yes, who recommended it to you? ....

19. Do you take any vitamin D supplements? □ Yes □ No

If yes, how much? ....

If yes, who recommended it to you? ....

20. Do you consume alcoholic beverages? □ Yes □ No □ Occasionally

If yes, what do you consume? ....

If yes, in what quantities? ....

21. Do you consume caffeinated beverages daily? □ Yes □ No

..........If yes, what do you consume? (e.g., coffee, green or black tea, energizer) ....

..........If yes, in what quantities? ....

22. Do you smoke? □ Yes □ No

If yes, how much? .... cigarettes per day

If yes, for how long? .... years

23. How much exercise do you do daily?

□ <30 min/day □ 30 min/day □ >30 min/day □ >60 min/day

What kind of physical activities do you practice (e.g., walk)? ....

24. You have any of the following pathologies (one or more)?

□ Diabetes mellitus

□ Kidney problems

□ Hyperthyroidism

□ Cancer (breast, prostate)

□ Rheumatoid arthritis

□ Ulcerative colitis

□ Crohn's disease

□ Celiac disease

□ Cushing Syndrome

□ Factors that can cause low estrogen or testosterone levels (e.g., anorexia or bulimia, excessive exercise, anti-tumour treatments)

□ Not the case

25. Regarding the surgical history, have you undergone any interventions? □ Yes □ No

If yes, what kind of interventions?

□ Thyroid surgery □ Spine surgery □ Hip surgery

□ Removal of the uterus

At what age? .... years

□ Removal of the ovaries: □ one □ both

At what age? .... years

□ Other types of surgeries: ....

26. Are you or have you ever been on treated with one of the following medications (for more than 3 consecutive months per year)?

□ Corticosteroids (Prednisone, Prednisolone, Hidrocortizone)

□ Anticonvulsants (Valproic acid, Carbamazepine, Pregabalin, Lamotrigine, Topiramate)

□ Anti-tumorals (Tamoxifen, Exemestane = Aromastin®/Xanepra®, Bicalutamide = Casodex®/Yonistib®)

□ Proton Pump Inhibitors (Omeprazole = Omez®, Omeran®, Esomeprazole = Nexium®/Controloc®)

□ Certain antidepressants (Venlafaxine = Fobiless®, Duloxetine = Cymbalta®/Dulsevia®, Escitalopram = Cipralex®)

□ Not the case

27. Do you have a family history of osteoporosis? □ Yes □ No

If yes, who suffers or has suffered from osteoporosis? ....

28. Has your general practitioner informed you of the risk of developing osteoporosis? □ Yes □ No

29. Are you currently diagnosed with osteoporosis? □ Yes □ No

30. Have you ever undergone a bone quality test? (DXA test/osteodensitometry) □ Yes □ No

If yes, how many times did you get tested? ....

If yes, when was the last time you got tested? ....

31. Are you currently under any treatment for osteoporosis? □ Yes □ No

a. If yes, what exactly?

□ Alendronic acid = Fosamax®/ Fosvance®

□ Risedronic acid-Actonel®

□ Zoledronic acid = Zometa®/ Aclasta®

□ Denosumab = Prolia®/ Xgeva®

□ Ibandronic acid = Bondronat®/Bonviva®

□ Calcitriol

□ Raloxifene = Evista®

□ Strontium ranelate

□ Hormone replacement therapy

□ Other osteoporosis treatment: ....

b. If yes, for how long did you have this treatment? ....

c. If yes, did you feel any improvements? □ Yes □ No

If yes, what exactly? ....

d. Have you ever experienced any adverse effects? □ Yes □ No

If yes, with what? ....

Thank you for taking the time to complete this questionnaire!
